# A compact two-electrode field-emission X-ray source for soft X-ray spectroscopy applications

**DOI:** 10.1107/S160057752501166X

**Published:** 2026-02-10

**Authors:** Junlin Li, Yicheng Wang, Pengfei Yu, Jianwei Meng, Tsuchien Weng

**Affiliations:** a Center for Transformative Science Shanghaitech University No. 393 Middle Huaxia Road Shanghai201210 People’s Republic of China; b School of Physical Science and Technology Shanghaitech University No. 393 Middle Huaxia Road Shanghai201210 People’s Republic of China; ESRF – The European Synchrotron, France

**Keywords:** field-emission X-ray sources, soft X-ray spectroscopy, XES, blade electrodes, SXFEL

## Abstract

Here, a compact two-electrode field-emission X-ray source employing a blade-type tungsten cathode was designed for soft X-ray spectroscopy. The device suppressed filament-derived optical and thermal background that is intrinsic to thermionic tubes, while providing stable and tunable output. The cathode–anode spacing was optimized and an optimal gap was identified at 150 µm, enabling the source to reach 400 µA at 4 kV without short circuits at <1 × 10^−7^ mbar. Spatial intensity mapping with targets tilted at 30°, 45° and 60° showed that smaller tilts yielded a more concentrated flux, with full width at half-maximum values of 28°, 54° and 76° at 2.5 kV, respectively, while the peak emission direction remained near 30° independent of the applied voltage and the tilt angle. Energy-resolved measurements with a silicon drift detector showed that the high-energy cutoff of the *Bremsstrahlung* continuum shifted linearly with the applied acceleration voltage, and that a reproducible Cu *L*α peak appeared at 927.7 eV, confirming stable and proper operation of the source. High signal-to-noise soft X-ray emission spectroscopy of Ti *L*α and O *K*α lines were obtained using the source integrated at the Ultrafast X-ray Spectroscopy (UXS) endstation of the Shanghai Soft X-ray Free-Electron Laser (SXFEL) facility. The blade cathode and simplified architecture improve manufacturability and cost efficiency, providing a laboratory-scale low-background source for element-specific soft X-ray spectroscopy and pre-beamline experiments.

## Abstract 2

Here, a compact two-electrode field-emission X-ray source employing a tungsten blade cathode was developed to deliver stable low-background output optimized for soft X-ray spectroscopy. The device’s capability for high signal-to-noise element-specific analysis was demonstrated through successful TiO_2_ emission spectroscopy at the SXFEL facility, offering a practical laboratory-scale alternative to synchrotron sources.

## 1. Introduction

Soft X-ray spectroscopy, including X-ray absorption spectroscopy and X-ray emission spectroscopy (XES), provides element-specific and orbital-selective insights that enable *operando* studies of catalytic processes, probe electronic correlations in energy materials, and support label-free bio-characterization (Liu *et al.*, 2019 ▸, 2020 ▸; Bonanni & Gianoncelli, 2023 ▸; Zhou *et al.*, 2023 ▸). These applications impose stringent requirements on the X-ray source, notably tunable photon energy, high photon flux for trace-element sensitivity, excellent temporal and long-term stability, and a very low optical background to preserve measurement fidelity.

High-quality soft X-ray spectroscopy is typically performed at large-scale facilities. Synchrotron radiation provides high-flux, monochromatic and stable X-rays (Cramer, 2017 ▸; Galayda, 1996 ▸). While X-ray free-electron lasers offer femtosecond pulses, high peak brightness and transverse coherence for ultrafast studies of chemical dynamics (Tschentscher, 2023 ▸; Schreiber & Faatz, 2015 ▸; Liu *et al.*, 2022 ▸). However, high capital and operating costs, together with limited beam time, constrain access and motivate complementary laboratory-scale solutions. Conventional laboratory X-ray tubes based on thermionic emission suffer from substantial visible and thermal radiation from the heated filament, which can obscure weak spectral features and degrade the signal-to-noise ratio even with optical filtering (William, 1968 ▸).

Field-emission (FE) X-ray sources address these limitations by extracting electrons via quantum tunnelling under strong electric fields, thereby eliminating filament heating and suppressing filament-derived optical background. FE cathodes also provide a fast response and high emission current density (Giubileo *et al.*, 2018 ▸; Pribat *et al.*, 2007 ▸). Building on these advantages, here we develop a compact two-electrode FE X-ray source engineered for soft X-ray spectroscopy. In this work, a tungsten blade is employed as the cathode. Compared with traditional needle-like tips or nanotube electrodes, the blade-type geometry provides a significantly larger effective emission area, which allows for higher total beam currents (and thus higher photon flux). Furthermore, the bulk structure of the blade offers superior thermal dissipation and mechanical robustness, effectively mitigating the ‘tip blunting’ effect caused by Joule heating and ensuring more stable operation under low emission voltages (Alivov & Molloi, 2010 ▸; Giubileo *et al.*, 2018 ▸). A two-electrode structure further simplifies fabrication compared with triode configurations. The source operates under ultrahigh vacuum with optimized cathode–anode spacing and target geometry to deliver adjustable photon flux with high stability and low optical background.

In this work, we quantify emission output and stability, evaluate the impact of cathode–anode spacing on source performance, map the spatial distribution of emission intensity, and record characteristic X-ray spectra. To assess spectroscopic capability in a practical setting, the source is integrated into the Ultrafast X-ray Spectroscopy (UXS) endstation at the Shanghai Soft X-ray Free-Electron Laser (SXFEL) facility, where soft XES of TiO_2_ is demonstrated. These results establish a laboratory-scale low-background soft X-ray source that complements large-scale facilities for high signal-to-noise spectroscopy.

## 2. Instrumentation

### 2.1. Structural design and emission characterization of a compact field-emission X-ray source

In this work, we developed a compact FE X-ray source implementing a blade-edge cathode architecture. The operational principle relies on field electron emission under high electric fields: upon application of a high voltage between the cathode and anode, the enhanced local electric field at the cathode blade induces quantum tunnelling of electrons. These emitted electrons form a focused beam that bombards a metal target (anode), producing characteristic and *Bremsstrahlung*X-rays. The FE process follows the Fowler–Nordheim (F–N) theory (Chiou *et al.*, 2001 ▸), where the current density *J* is expressed as
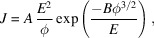
where *E* represents the local electric field, ϕ is the cathode work function, while *A* and *B* are material-specific constants. Notably, the exponential field dependence makes emission extremely sensitive to cathode geometry and applied voltage.

**Figure 1:**
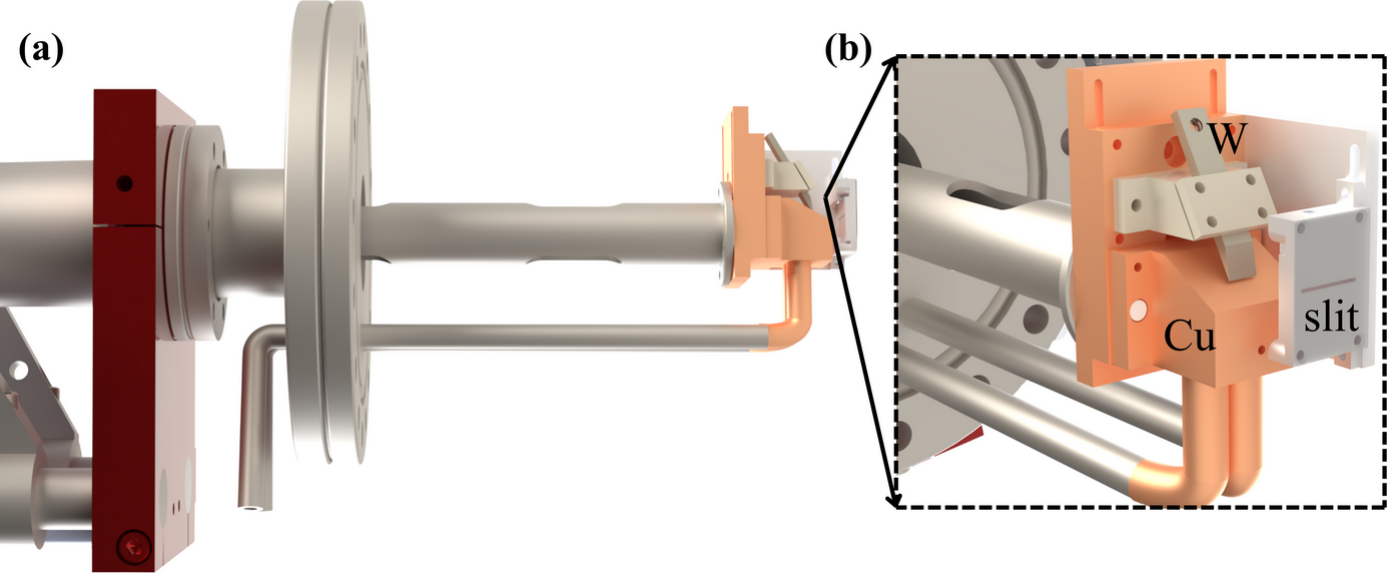
(*a*) Schematic diagram of the FE X-ray source showing the overall configuration of the cathode and anode. (*b*) Enlarged view of the cathode–anode region showing the structural details of the emission area.

Fig. 1 ▸ illustrates the experimental configuration housed in a high-vacuum chamber (base pressure: 9.6 × 10^−8^ mbar). To meet spectroscopic requirements for high beam intensity, we implemented an innovative blade-type cathode design. This geometry creates an equivalent array of emission sites, significantly enhancing the local field enhancement factor (β) compared with conventional needle-like emitters. The anode consists of an oxygen-free copper block machined with a trapezoidal profile to optimize X-ray take-off angle and detection efficiency. The power-supply configuration employs negative high-voltage biasing of the cathode with grounded anode, ensuring both operational safety and minimized electromagnetic interference.

The FE X-ray source’s performance was quantified through emission power characterization and stability analysis. As predicted by F–N theory, emission current exhibits strong nonlinear dependence on applied voltage *V* (∝ *E*). We systematically increased *V* from 3 to 8 kV in 0.5 kV increments while monitoring transient current response. X-ray output power was derived from current–voltage (*I*–*V*) measurements and target conversion efficiency. Long-term stability was assessed through continuous operation at optimal voltage, with real-time vacuum monitoring via a calibrated full-range gauge, which enabled systematic evaluation of the influence of vacuum fluctuations on emission power and analysis of the potential impact of surface adsorption/desorption processes on cathode stability.

### 2.2. The emission characteristics of field-emission X-ray sources

According to the classical F–N FE theory, the emission-current density thus exhibits an exponential dependence on the local electric field. And the electric field is closely related to the cathode–anode spacing, which directly affects the emission power of electrons, which in turn affects the emitted power of the X-rays. Optimization of the cathode–anode spacing is consequently essential for achieving a high-brightness and stable X-ray source.

**Figure 2:**
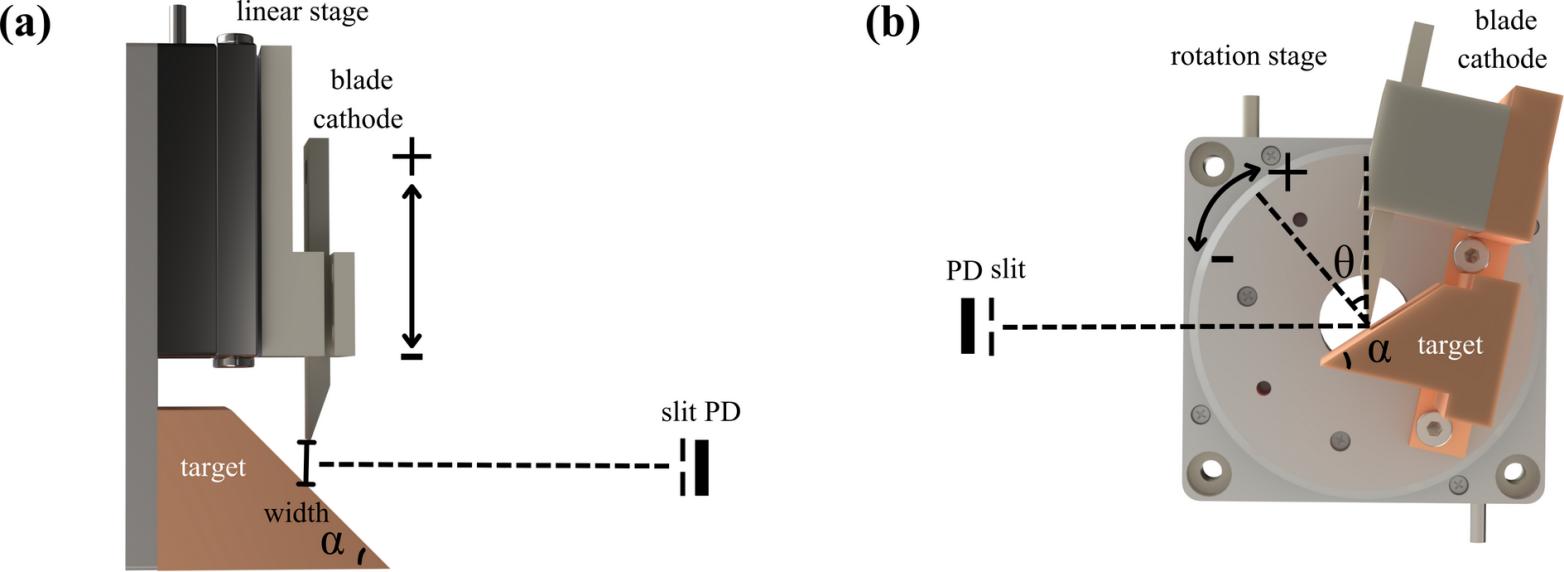
Schematic diagram of the experimental setup for measuring (*a*) the influence of cathode–anode spacing and (*b*) the spatial intensity distribution.

To investigate the effect of the cathode–anode spacing on emission characteristics, a dedicated experimental setup was constructed [Fig. 2 ▸(*a*)]. The cathode was mounted on a PEEK insulating holder providing mechanical rigidity and electrical isolation, while the anode consisted of a copper target combined with a photodiode (AXUV-100G) for measuring the intensity of emitted X-ray from the target. The cathode–anode spacing was adjusted using an ultra-high vacuum linear piezo stage with submicrometre positioning accuracy. All components were installed inside a stainless-steel vacuum chamber supported by a rigid mechanical frame to ensure long-term stability and to maintain a base pressure below 10^−7^ mbar

During the measurements, the spacing between the cathode and anode was varied continuously from 50 to 500 µm under a constant accelerating voltage. The emission current and corresponding X-ray output power were recorded at each distance, and the fluorescent screen was used to monitor the spatial uniformity and temporal stability of the emission. This configuration allowed systematic characterization of the source performance as a function of electrode spacing and identification of the optimal geometry for steady X-ray generation.

It is well established that X-ray radiation generated by electron bombardment of metal targets exhibits non-uniform spatial intensity distribution. To achieve optimal X-ray flux, we aimed to concentrate the radiation within a narrow angular range. This concentration strategy effectively confines the limited number of photons to a smaller detection area, thereby significantly enhancing the X-ray flux output.

To implement this approach, we mounted an FE X-ray device on a high-precision piezoelectric rotation stage. The emission point was carefully aligned with the stage’s rotational centre through precise calibration to ensure position stability during operation. The experimental configuration is illustrated in Fig. 2 ▸(*b*).

For intensity measurement, a photodiode detector was positioned in front of the X-ray emission point, and the distance was fixed to 63 mm. To guarantee measurement precision, a calibrated slit was installed in front of the photodiode, restricting detection to a well defined small solid angle consistent with our target angular range. Theoretically speaking, the spatial distribution of light intensity measured by a stationary photodiode while rotating an FE X-ray source is equivalent to rotating a photodiode while keeping the light source stationary.

Due to experimental constraints, we examined the relationship between target angle [α shown in Fig. 2 ▸(*b*)] and X-ray intensity distribution using targets with fixed inclination angles of 30°, 45° and 60°. This comparative study systematically analysed how target-angle parameters influence radiation intensity distribution.

### 2.3. Energy distribution and spectroscopic characteristics of the X-ray source

**Figure 3:**
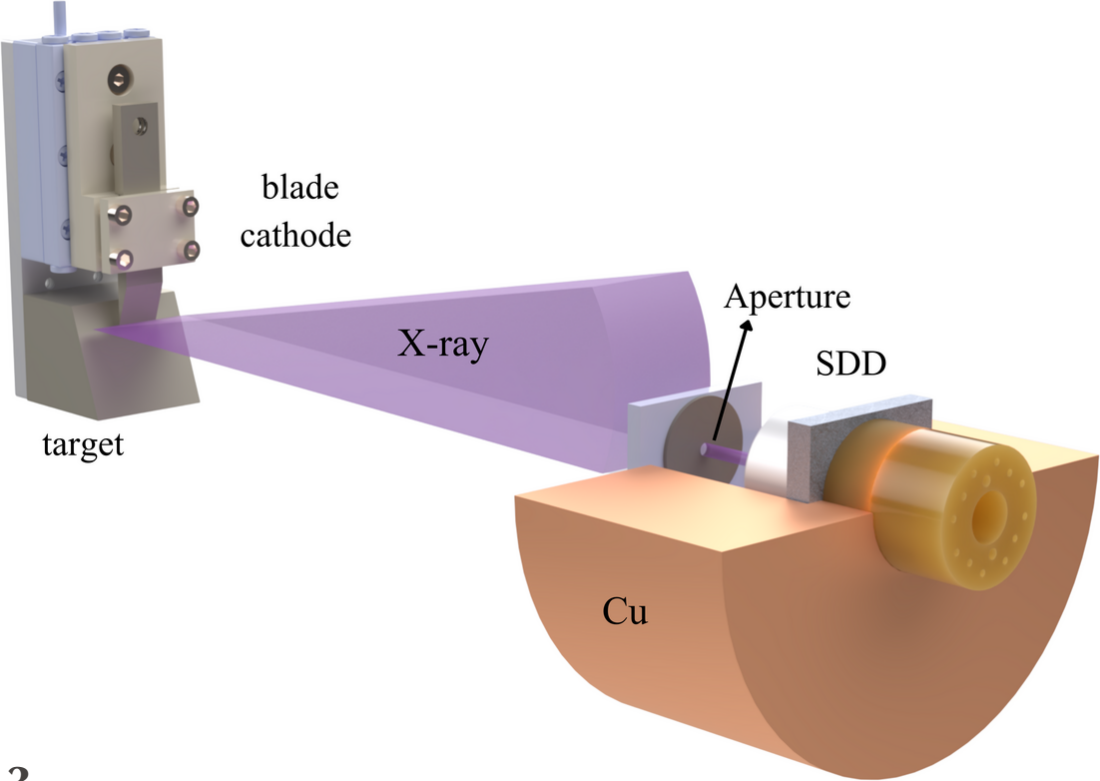
Schematic diagram of the experimental setup for measuring the X-ray energy distribution. The SDD is positioned 400 mm away from the X-ray source to record the photon energy spectrum.

To investigate the emission characteristics of the FE X-ray source, we measured its photon energy spectrum using a silicon drift detector (SDD). The experimental setup is illustrated in Fig. 3 ▸. The X-ray source consists of a blade-type cathode and a target. The cathode was mounted on a high-precision linear stage to adjust the blade-to-target distance, with electrical isolation provided by a PEEK insulator. High-purity titanium was employed as the target material, whose characteristic *K*α line energy (4.512 keV) falls within the medium-energy range suitable for SDD measurements. To prevent photon-flux saturation and reduce dead-time effects in the SDD, X-ray intensity was attenuated using an appropriately thick polyimide film. Additional flux reduction was achieved by installing a 400 mm-long straight tube between the source and detector, along with a collimator positioned ∼10 mm before the detector to maintain photon count rates within the detector’s linear response range.

Following optimization of the cathode–anode gap and spatial intensity distribution, this study examined the dependence of the X-ray energy spectrum on accelerating voltage. Stepwise voltage scans from 3 to 5 kV were performed, with the SDD recording photon energy distributions in real time. These measurements established the relationship between characteristic X-ray spectra and accelerating voltage in the FE source, laying the groundwork for its future application in X-ray spectrometers.

### 2.4. Energy-spectrum characteristics of the field-emission X-ray source in the soft X-ray range

**Figure 4:**
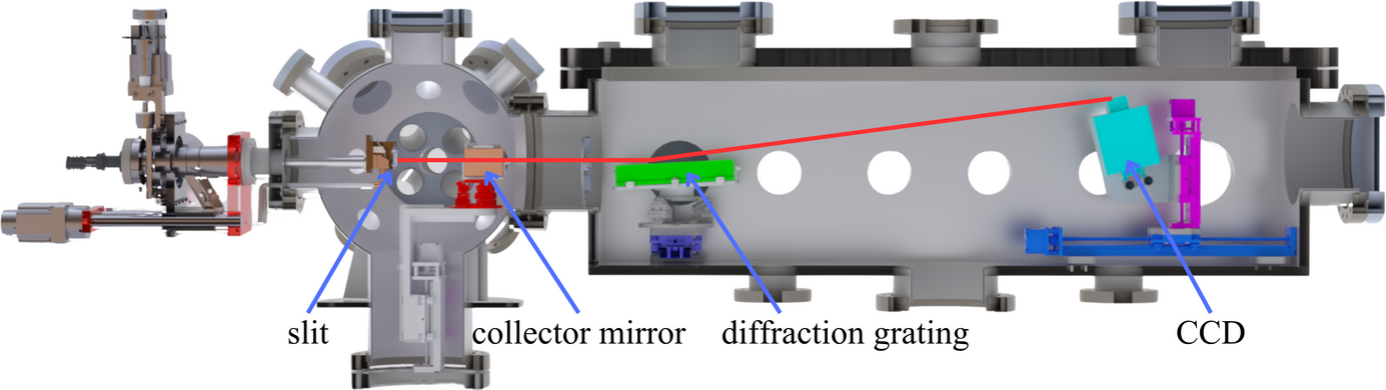
Schematic diagram of the spectroscopic experimental setup. The FE X-ray source is installed at the UXS endstation and its position is precisely adjusted to align with the beamline geometry for spectroscopic measurements.

To obtain the characteristic energy spectrum of the FE X-ray source in the soft X-ray energy range, we installed the device at the UXS endstation, as illustrated in Fig. 4 ▸. This station, one of the experimental endstations at the SXFEL facility, is equipped with a high-resolution soft X-ray emission spectrometer. The spectrometer primarily consists of a collection mirror, an X-ray grating, a vacuum back-illuminated CCD detector and motorized adjustment mechanisms, and operates within a vacuum environment. The collection mirror is an elliptical cylindrical mirror focusing the spot vertically. The X-ray grating, an equidistant cylindrical grating with a line density of 900 lines mm^−1^, covers an energy measurement range of 250–750 eV and achieves an energy resolution of up to 2000 at 400 eV. The detector utilizes a CCD sensitive to direct X-ray detection, offering high sensitivity for soft X-rays, enabling the measurement of weak sample signals.

A fixed slit measuring 20 µm × 1 mm was placed in the sample point, which was positioned on the same Rowland circle with the grating and CCD detector. Then the FE X-ray source was placed behind the slit, simulating the narrow line fluorescence signal emitted by the sample.

To improve heat dissipation from the target and maintain stable vacuum conditions inside the chamber, a water-cooling system was added to the rear side of the copper target. The circulating coolant efficiently removes the heat generated by electron bombardment, reducing the accumulation of heat on the target surface and improving the stability of emission power. These modifications provide a thermally stable working environment that benefits subsequent spectroscopic measurements.

## 3. Results and discussion

### 3.1. Verification of field-emission behaviour and emission stability

**Figure 5:**
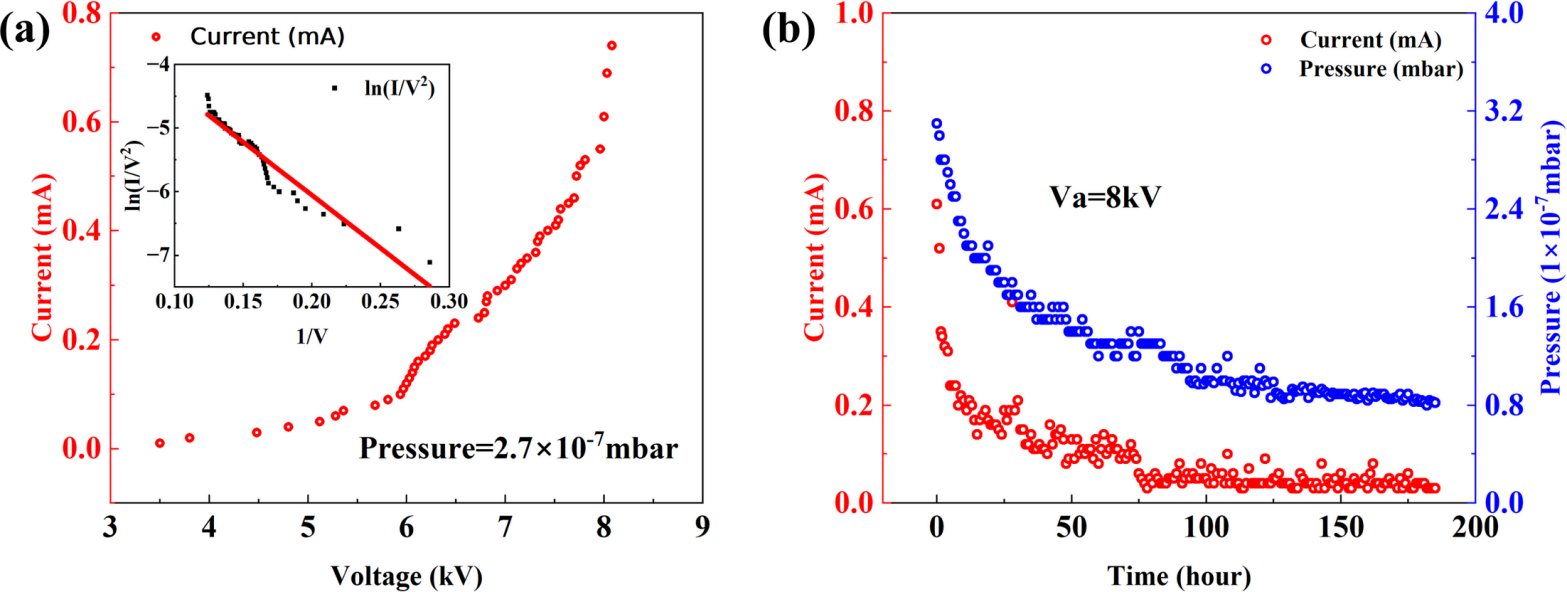
(*a*) Measured emission power of the FE X-ray source under a base pressure of 2.7 × 10^−7^ mbar and (*b*) the stability test performed at an input voltage of 8 kV.

To gain deeper insights into the emission characteristics of blade-type FE X-ray sources, we conducted a systematic investigation. First, the relationship between emission voltage and current was examined, as shown in Fig. 5 ▸(*a*). It can be observed that the emission current exhibits exponential growth with increasing voltage. According to the F–N FE formula, the relationship between emission current *I* and applied voltage *V* can be expressed as (Chiou *et al.*, 2001 ▸; Forbes, 2008 ▸)
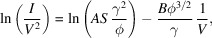
where *A* and *B* are constants with values of *A* ≃ 1.541434 × 10^−6^ A eV V^2^ and *B* ≃ 6.83089 × 10^9^ eV^−3/2^ V m^−1^, and *S* corresponds to the area of FE current. Here, we provide a rough estimation without considering corrections for barrier shape or other detailed analyses. For simplicity, the relationship between electric field strength *E* and voltage *V* is expressed as
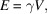
where γ is the electric field-to-voltage conversion factor.

A linear fit of this relationship allows us to derive the work function ϕ and the slope *M*. From the linear fitting of Fig. 5 ▸(*a*) (inset), the slope *M* = *B*ϕ^3/2^/γ = −16676.7 V was obtained. Substituting this into the formula yields the relationship between ϕ and γ. The conversion factor γ, which depends on the geometric configuration of the FE X-ray source, was calculated for a blade made of high-purity tungsten with a length of 5 mm and a tip curvature radius *r* ≃ 10^2^ nm. The system was approximated as a cylindrical electrode of radius *r* and an infinite planar electrode. The maximum electric field strength between them is given by
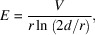
where *d* is the distance between the cylindrical and planar electrodes. Thus, γ can be expressed as
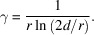
Using *r* ≃ 100 nm and *d* = 150 µm, we obtained γ ≃ 1.24 × 10^6^ m^−1^. Substituting this value into the equation, the work function of the FE electrode was calculated as ϕ ≃ 2.1 eV. This value is significantly lower than the well known work function of tungsten (∼4.5 eV). Furthermore, the linear fit between ln (*I*/*V*^2^) and 1/*V* was not ideal, with a coefficient of determination of 0.91334.

We attribute this discrepancy to the relatively high background pressure in the vacuum environment, which causes emitted electrons from the blade to collide with gas molecules, leading to ionization and the generation of additional electrons. These electrons are further accelerated by the electric field and eventually bombard the target material, producing characteristic and *Bremsstrahlung* radiation. Meanwhile, the resulting ions migrate toward the blade electrode, contributing to an ion current whose magnitude is directly related to the vacuum level. Generally, the number of ions generated per unit time is approximately proportional to the gas-molecule density (Jousten *et al.*, 2020 ▸). Therefore, under poor background vacuum conditions, the emission-current characteristics resemble those of the background pressure data measured by the vacuum gauge.

To verify this, we simultaneously recorded the temporal variations in the FE current and the vacuum-gauge readings, as shown in Fig. 5 ▸(*b*). When the emission voltage was held constant (Va = 8 kV), the FE current and background pressure exhibited similar trends over time. After ∼200 h of continuous measurement, the background pressure approached 8 × 10^−8^ mbar and the emission current decreased to the microampere range. These results indicate that the primary contribution to the emission current originates from the ionization of gas molecules in the background vacuum. Only when the background pressure is sufficiently low does the tunnelling current dominate. To some extent, a higher background pressure is advantageous for increasing emission power due to the ion current generated by gas ionization. However, when the gas-molecule density becomes excessively high, the electrons produced by ionization are accelerated by the electric field and bombard additional gas molecules, creating a cascade effect that rapidly amplifies the current and may lead to short-circuiting.

### 3.2. Influence of cathode–anode spacing on emission performance and operational stability

Improving the base vacuum reduces the probability of gas-molecule ionization, thereby lowering the incidence of cascade-induced short circuits between electrodes and enhancing the operational stability of FE devices. Electrode spacing is another key determinant of stability because it directly shapes the internal electric field and the resulting emission current. As the gap increases, the electric field decreases in an exponential manner, leading to a sharp reduction in emission current; conversely, narrowing the gap strengthens the field and markedly increases the current, but also elevates the likelihood of gas ionization and short circuits, ultimately degrading stability.

**Figure 6:**
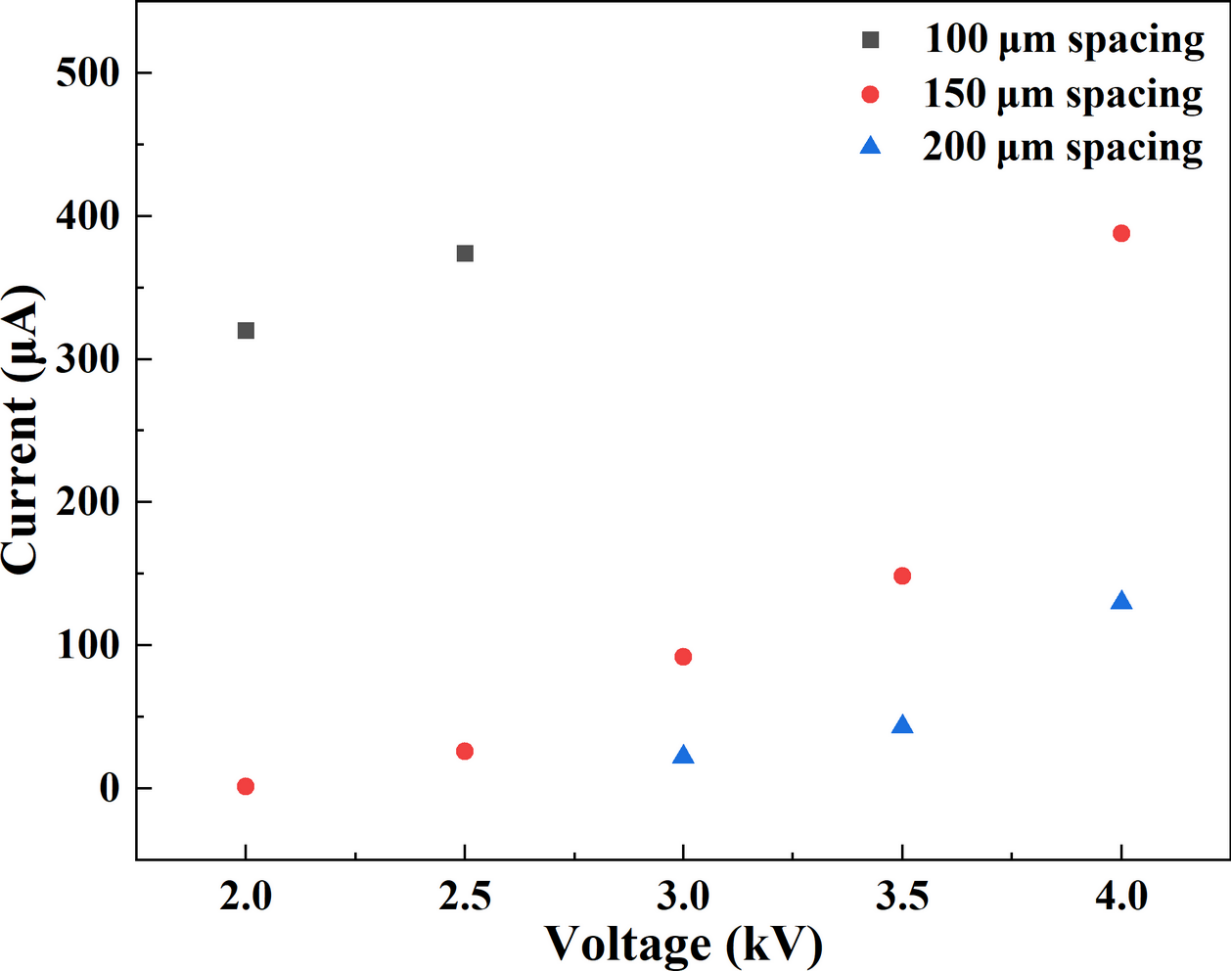
Measurement results of the FE X-ray source at different cathode–anode spacing under a base pressure of 9 × 10^−8^ mbar.

To systematically evaluate device stability, we investigated the emission characteristics at a fixed base pressure of 9 × 10^−8^ mbar for different cathode–anode gaps (Fig. 6 ▸). With a 100 µm gap, the emission current exceeds 300 µA at 2 kV and rises rapidly with further increases in voltage; however, pronounced short circuits appear above 2.5 kV, preventing stable operation at higher voltages. When the gap is increased to 150 µm, the current at 2 kV is two orders of magnitude lower than in the 100 µm case, yet it grows steadily with voltage and reaches 400 µA at 4 kV, with no short circuits observed throughout, indicating robust stability. At a 200 µm gap, 2 kV is insufficient to reach the threshold for FE; a microampere-level current appears at 3 kV and exceeds 100 µA at 4 kV. While stability is essentially comparable to the 150 µm case, the available current is only about one quarter.

In summary, under a base pressure of 9 × 10^−8^ mbar and for operating voltages below 4 kV, an electrode gap of 150 µm offers the most favourable balance, maintaining stable operation while delivering the highest emission current. For stable operation at higher voltages, increasing the electrode gap or further reducing the base pressure are both effective strategies to suppress gas ionization and mitigate short-circuit events.

### 3.3. Analysis of spatial intensity distribution of the field-emission X-ray source

The spatial emission characteristics of the FE X-ray source were systematically investigated under varying accelerating voltages and target tilt angles [α in Fig. 2 ▸(*b*)]. At each fixed voltage, three sets of intensity data were recorded at every angular position with a 5 s interval. The averaged values were used to minimize random noise and transient fluctuations.

**Figure 7:**
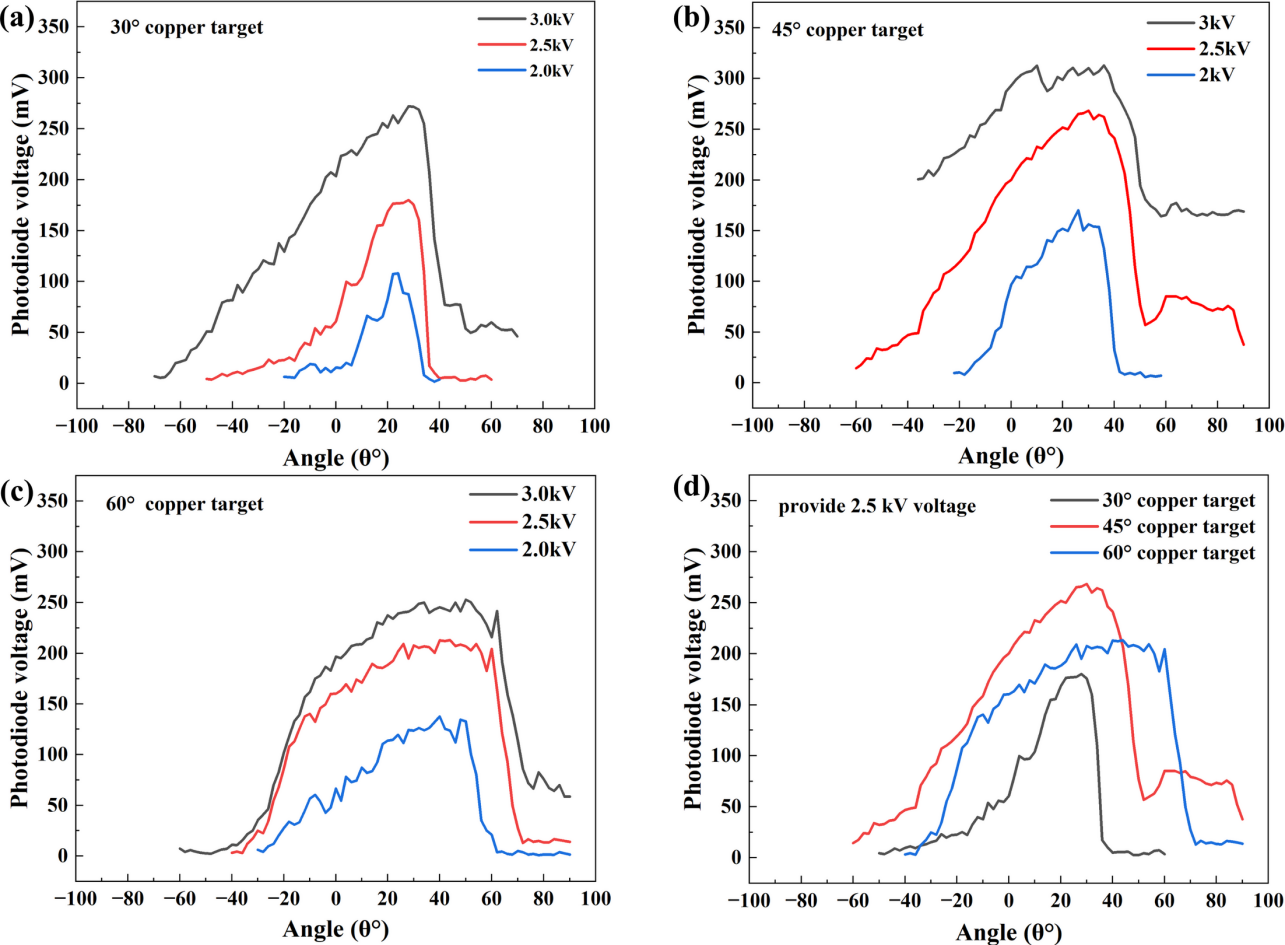
Spatial intensity distributions of the FE X-ray source measured with different target tilt angles: (*a*) 30°, (*b*) 45° and (*c*) 60° Cu targets. (*d*) Comparison of the spatial intensity distributions of the three Cu targets measured at an accelerating voltage of 2.5 kV.

Figs. 7 ▸(*a*)–7 ▸(*c*) show the spatial distributions of the FE X-ray intensity for target tilt angles of 30°, 45° and 60°, respectively. When the target tilt is 30°, the intensity exhibits a pronounced left–right asymmetry. This arises from the target geometry, which restricts emission on the positive-angle side. As the tilt angle increases, this asymmetry weakens. When the applied voltage is raised from 2 to 3 kV, the overall intensity increases and the full width at half-maximum (FWHM) broadens markedly, while the position of the intensity peak remains essentially unchanged. The same behaviour persists as the tilt angle is increased from 30° to 60°. However, at larger tilt angles the voltage-induced change in the FWHM becomes less pronounced. The position of the peak is largely independent of the applied voltage; in other words, the magnitude of the voltage does not affect the spatial location of the intensity maximum.

To further clarify how the spatial distribution depends on the target tilt, we fixed the emission voltage and varied the tilt angle, then measured the corresponding intensity distributions; the results are shown in Fig. 7 ▸(*d*). At an emission voltage of 2.5 kV, the FWHM values are 28°, 54° and 76° for tilt angles of 30°, 45° and 60°, respectively. This indicates that as the tilt angle increases, the spatial distribution becomes more uniform. Such uniformity is unfavourable for X-ray spectroscopic applications that seek higher photon counts, because a more uniform distribution yields fewer photons per unit solid angle along the direction of maximum intensity. To maximize photon flux along the peak-intensity direction, the target tilt should be kept relatively small. Throughout these variations (whether changing the emission voltage or the tilt angle) the position of the intensity maximum remains nearly constant, at ∼30°. This behaviour indicates that the angular distribution of *Bremsstrahlung* generated by electron-beam deceleration depends solely on the electron-beam direction (Omar *et al.*, 2018 ▸, 2020 ▸). Consequently, parameters that do not alter the beam direction, such as the target tilt and the acceleration voltage, do not shift the direction of the maximum *Bremsstrahlung* intensity.

### 3.4. Analysis of the spectral characteristics of the field-emission X-ray source

**Figure 8:**
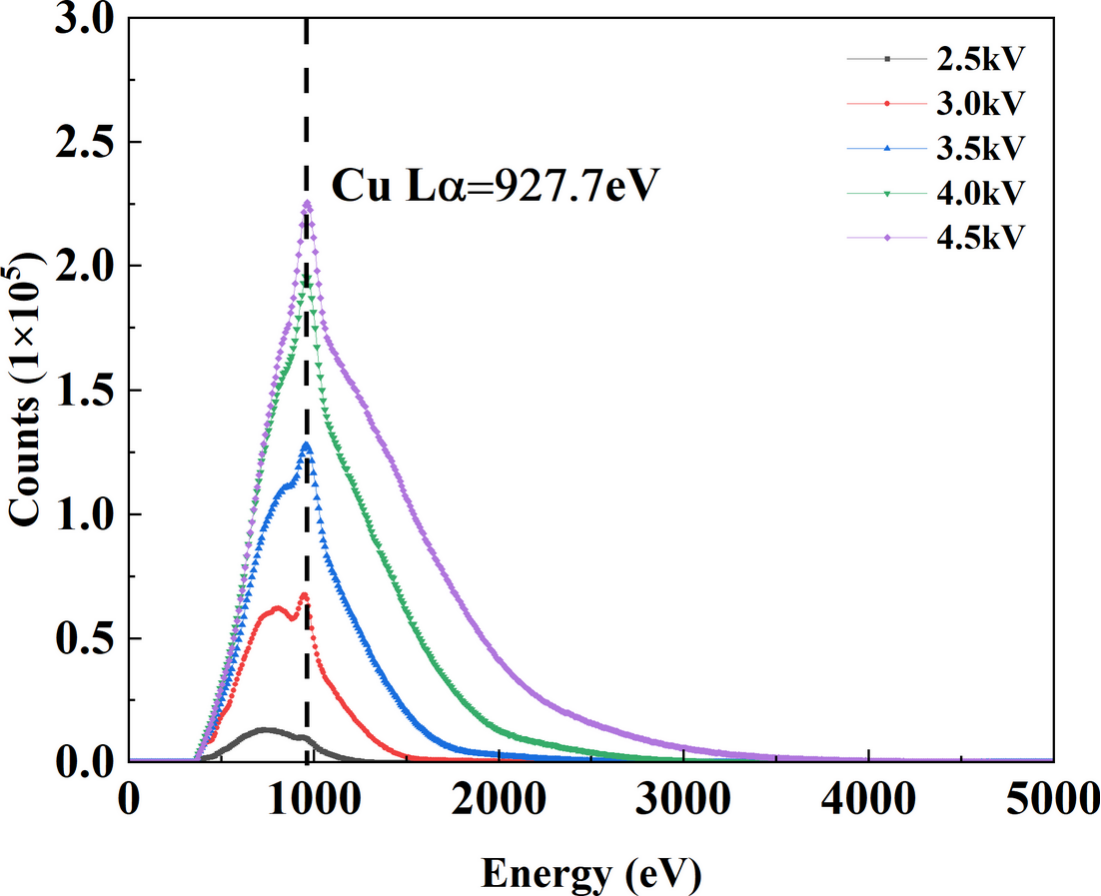
Measured energy distribution of the FE X-ray source.

To characterize the energy spectrum of the FE X-ray source, we measured its emission with an SDD equipped with a 50 µm beryllium window at accelerating voltages of 2.5–4.5 kV (Fig. 8 ▸). At all voltages, the spectra comprise a broad *Bremsstrahlung* continuum superimposed with Cu *L*-series characteristic radiation. The Cu *L*α peak remains fixed at 927.7 eV, indicating a stable and accurate energy calibration with no appreciable drift. The high-energy endpoint of the continuum shifts approximately linearly with voltage and decays to zero near the maximum voltage *U*, consistent with the thick-target *Bremsstrahlung* endpoint relation. The total counts increase markedly with voltage, with the enhancement of the *L* lines outpacing that of the continuum. At 2.5 kV the overall intensity is weak and the *L* peak is not prominent, whereas at 4.5 kV the Cu *L* peak clearly rises above the local background. This differential enhancement can be attributed to the overvoltage factor (*f* = *eU*/*E*_edge_, where *U* is the maximum applied voltage, *E*_edge_ the absorption edge of the target and *e* the charge of an electron) (Meisenkothen *et al.*, 2009 ▸); raising the tube voltage increases the overvoltage factor, which drives a rapid increase in the *L*-shell ionization cross section and thus the characteristic yield. Concurrently, higher voltage typically entails a larger electron dose and greater average energy deposition in the target, which elevates the continuum intensity.

Instrument response significantly shapes the observed spectra. First, the SDD’s limited energy resolution near 1 keV prevents resolution of the fine structure (*e.g.L*α and *L*β of copper), which appears as a single broadened feature. Second, the 50 µm Be window has poor transmission for soft X-rays, suppressing counts in the few-hundred-eV range and causing a systematic underestimation at the low-energy end. Overall, the spectra exhibit a controllable evolution with voltage, and strong Cu *L* characteristic emission centred near 1 keV is obtained in the 4.0–4.5 kV range.

### 3.5. Spectroscopic characterization of the field-emission X-ray source in the soft X-ray region

To obtain high-resolution spectral features of the FE X-ray source in the low-energy range (<900 eV), we integrated the device into the UXS endstation of the SXFEL facility and performed energy-resolved measurements using its high-resolution soft X-ray emission spectrometer. Because a copper target lacks elemental characteristic lines in this energy range, we evaporated an ∼500 nm TiO_2_ thin film onto the copper substrate to generate Ti *L* and O *K* characteristic radiation, enabling comparison between characteristic emission and *Bremsstrahlung*. The film thickness was chosen by balancing the soft X-ray absorption length in TiO_2_ against the electron-beam excitation volume, thereby effectively suppressing contributions from the copper substrate within the measurement window.

**Figure 9:**
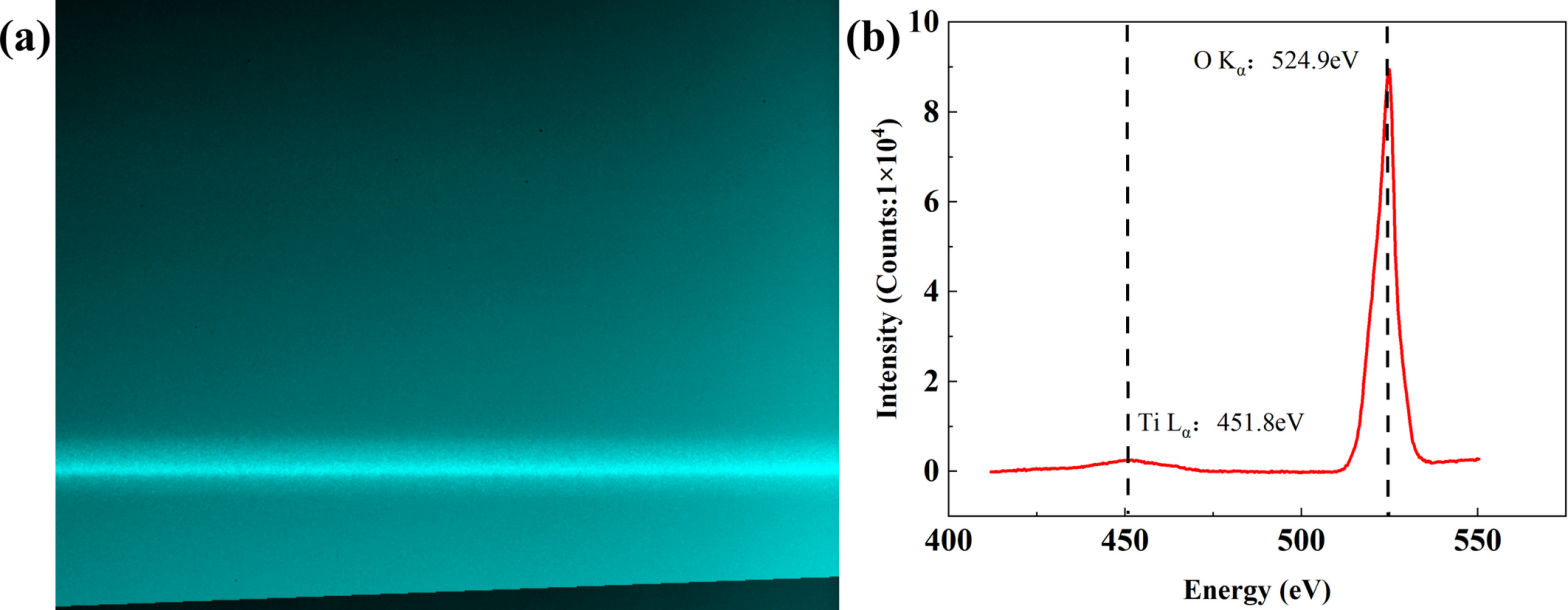
(*a*) Raw CCD image of the emission signal and (*b*) X-ray emission spectrum of TiO_2_ measured at the UXS endstation of the SXFEL.

Fig. 9 ▸ shows the emission spectrum of the FE X-ray source over 400–560 eV. Two dominant lines are observed at 451.8 eV (Ti *L*α) and 524.9 eV (O *K*α). The measured peak positions agree with reported values for TiO_2_ (Ti *L*α ≃ 452 eV, O *K*α ≃ 525 eV), confirming the reliability of the energy calibration. In terms of intensity, the O *K*α peak is markedly stronger than the Ti *L*α peak; estimates from the peak heights indicate at least an order-of-magnitude difference. This disparity can be understood from the combined effects of the material’s electronic structure and the excitation–emission process: in TiO_2_, the valence band is dominated by O 2*p* states, while Ti^4+^ is near a *d*0 configuration with few occupied 3*d* states, resulting in relatively sparse radiative recombination channels for a Ti 2*p* core hole. In addition, the O:Ti stoichiometry of 2:1 leads to an O 2*p*-dominated valence band, further enhancing the O *K*α emission intensity.

The O *K*α feature exhibits a relatively sharp main peak with additional broadening arising from the finite valence-band width; its FWHM is ∼10 eV, consistent with the energy broadening associated with filling an O 1*s* core hole from the broad O 2*p* valence band. By contrast, the Ti *L*α signal is significantly weaker and broadened, attributable to strong 2*p*–3*d* Coulomb interactions, crystal-field splitting and ligand-to-metal charge transfer. This broadening behaviour is consistent with reported soft X-ray emission spectra of TiO_2_, further corroborating the oxidation state and chemical environment of the film (Terauchi *et al.*, 2018 ▸).

The continuous background in the low-energy region arises primarily from *Bremsstrahlung* generated by electron-beam bombardment of the target; however, its magnitude in the present energy range is low and does not hinder identification of the characteristic peaks. Taken together, these results demonstrate that the FE X-ray source can stably generate elemental characteristic radiation in the soft X-ray regime, and that the line energies and relative intensities are sensitive to the material’s chemical state and electronic structure. By selecting functional films as the target and controlling the film thickness, low-energy spectroscopic characterization of the films can be characterized. This provides an experimental basis for subsequent FE X-ray sources combined with soft X-ray spectrometers for soft X-ray spectroscopy measurements of samples.

## 4. Conclusions

In this study, we developed a compact FE X-ray source with a blade-type cathode design, optimized for soft X-ray spectroscopy applications. The device demonstrated high emission stability, tunable photon flux and significantly reduced optical background compared with conventional thermionic X-ray tubes. Systematic characterization revealed that a cathode–anode spacing of 150 µm and a base vacuum pressure below 1 × 10^−7^ mbar provided the optimal balance between emission current and operational stability. The spatial intensity distribution of the X-ray output was highly dependent on the target tilt angle, with smaller angles (*e.g.* 30°) yielding higher photon flux in the peak emission direction. However, the peak position is independent of the target’s tilt angle and applying voltage. The spectral measurements confirmed the generation of characteristic X-rays (Cu *L*α, Ti *L*α and O *K*α) with energies consistent with theoretical predictions, validating the source’s suitability for element-specific spectroscopy.

Integration of the FE X-ray source into SXFEL’s UXS endstation enabled high-resolution soft XES of TiO_2_, highlighting its potential as a laboratory-scale alternative to synchrotron facilities. The blade cathode design, combined with a simplified two-electrode structure, offers advantages in manufacturability and cost efficiency, making it a practical tool for preliminary experiments and routine measurements. Future work will focus on further improving emission uniformity and extending the operational lifetime, as well as exploring applications in time-resolved studies and *operando* spectroscopy.
